# Total Protein Profile and Drug Resistance in *Candida albicans* Isolated from Clinical Samples

**DOI:** 10.1155/2016/4982131

**Published:** 2016-07-11

**Authors:** Kamal Uddin Zaidi, Abin Mani, Vijay Thawani, Arti Mehra

**Affiliations:** Biotechnology Pharmacology Laboratory, Centre for Scientific Research & Development, People's University, Bhopal 462037, India

## Abstract

This study was done to assess the antifungal susceptibility of clinical isolates of* Candida albicans* and to evaluate its total protein profile based on morphological difference on drug resistance. Hundred and twenty clinical isolates of* C. albicans* from various clinical specimens were tested for susceptibility against four antifungal agents, namely, fluconazole, itraconazole, amphotericin B, and ketoconazole. A significant increase of drug resistance in clinical isolates of* C. albicans* was observed. The study showed 50% fluconazole and itraconazole resistance at 32 *μ*g mL^−1^ with a MIC_50_ and MIC_90_ values at 34 and 47 and 36 and 49 *μ*g mL^−1^, respectively. All isolates were sensitive to amphotericin B and ketoconazole. The SDS-PAGE protein profile showed a prevalent band of ~52.5 kDa, indicating overexpression of gene in 72% strains with fluconazole resistance. Since the opportunistic infections of* Candida* spp. are increasing along with drug resistance, the total protein profile will help in understanding the evolutionary changes in drug resistance and also to characterize them.

## 1. Introduction

Most of the common localised fungal infections are caused by* Candida *spp. The intensive use of antibiotics made these fungi more drug resistant and a clinical problem. Azoles are considered as the first level of management in* Candida* infection but there is increase in the resistance [1]. Since* C. albicans* is the common fungal causative agent in superficial and deep seated candidiasis [2, 3] and there has been rise in the incidence of antifungal resistance over the past decade [4, 5], it is important to evaluate the development of resistance pattern. Method of typing the organism based on the total protein expressed is useful in characterizing and understanding the development of drug resistance. Analysis of the proteins detects the genetic expression, which is an effective method for characterizing the species based on the morphological changes. The modification of the molecular morphology is a key marker to shift in the drug resistance and thus the protein profile helps in evaluating the antifungal resistance. The SDS-PAGE has been reported for epidemiological typing of* Klebsiella* spp. [6], aspergillosis [7], and* C. albicans* [8]. Ying et al. [9] indicated that the fluconazole resistance of* C. albicans* has overexpression of ERG11 gene. Keeping these findings in mind, this study was planned to find the incidence of* C. albicans* resistance to antifungal agents and to analyze their antifungal susceptibility pattern and characterize these isolates on the total protein content on their antifungal resistance.

## 2. Materials and Methods

### 2.1. Specimen

 All 120 clinical isolates of* C. albicans *from catheter tip (CT), urine (U), and high vaginal swab (HVS) were collected from the Department of Microbiology, People's College of Medical Sciences and Research Centre (PCMS and RC), Bhopal, during a month. The culture of* C. albicans *was maintained in the Sabouraud dextrose agar medium. The culture characterizes and microscopic observation was conducted to confirm the strains.

### 2.2. Antifungal Susceptibility

Well diffusion antifungal susceptibility assay was conducted based on the methodology of Magaldi et al. [10]. Emulsified colonies in the saline solution were inoculated in SDA medium in which wells were made using sterilized cork borer. After the moisture got absorbed, the antifungal agents fluconazole (20 *μ*g·mL^−1^), itraconazole (20 *μ*g·mL^−1^), amphotericin B (100 units), and ketoconazole (20 *μ*g·mL^−1^) were loaded into the well, and it was incubated at 37°C for 48 hrs.

### 2.3. Microdilution Assay

The minimal inhibitory concentration (MIC) of the standard drug, fluconazole, itraconazole, ketoconazole, and amphotericin B was determined by microdilution assay by culturing the fungi in Sabouraud dextrose broth and incubating them at 37°C for 48 hrs. The 96-well microtitre plates were prepared by dispensing 100 *μ*L of Sabouraud dextrose broth into each well and 100 *μ*L of the antifungal agents in a series from 0.5 to 64 *μ*g·mL^−1^. About 100 *μ*L of inoculum was then added to all the wells. The plates were then covered with Parafilm and incubated at 37°C for 48 hrs. All the procedures were conducted under sterile conditions. The MIC was determined by observing the colour change in the wells after addition of 3-(4,5-dimethylthiazol-2-yl)-2,5-diphenyltetrazolium bromide (MTT) which was defined as the lowest concentration that showed no growth.

### 2.4. Protein Profiling

To evaluate the total protein content the antifungal resistant strains were cultured in media with respective antifungal agents and total protein was extracted and the control was cultivated without antifungal agents. 5 g of pellet was collected by centrifuging at 5000 ×g for 10 min and washed twice in normal saline. To these pellets, 100 *μ*L of 10x Tris-glycine buffer (Tris 30.3 g, Glycine 144.2 g, SDS 10 g, and distilled water 1 liter: pH +8.3) and 10% SDS were added and sonication was performed in 4 bouts of 30 sec each in ice. After sonication, the supernatant was collected by centrifuging at 5000 ×g for 10 min at 4°C and stored at −20°C. The total protein profiling was done by resolving 10 *μ*g·*μ*L^−1^ total protein in 10% sodium dodecyl sulphate polyacrylamide gel electrophoresis [11]. Electrophoresis was performed at a 125 V for 4 h in running buffer of pH 8.3. After electrophoresis, the gels were stained with Coomassie Brilliant Blue R-250. The standards were used to make a plot of log molecular weight versus mobility of the wide range protein ladder (Himedia).

## 3. Results

*C. albicans *isolated from 120 infected cases had 51 high-risk patients. The major isolation source was catheterized patients (47.5%) and others were urine and high vaginal swab. The sources were from the age group 13 to 78 years. It was observed that more women had* Candida* infection with an infection ratio of 3 : 8 (M : W) with 71.43% UTI being higher in women. The isolates showed high sensitivity to amphotericin B and ketoconazole (100%) whereas 35% strains had itraconazole resistance and 50% showed fluconazole resistance. It was observed that the urine isolates had 23.26% of itraconazole and 13.95% of fluconazole resistance while the catheter sample showed 38.60% and 15.79% resistance, respectively. In men 30.77% itraconazole resistance and 33.33% fluconazole resistance were observed, whereas isolates from women had 32.79% and 36.07% resistance, respectively, indicating a similar antibiotic resistance in both genders. 64.17% itraconazole and fluconazole resistance were observed, of which 11 (14.29%) strains showed resistance to both antifungal agents. The total antifungal susceptibility has been shown in Table 1.

Comparison of MIC pattern (Table 2) of strains showed lowest MIC with amphotericin B and ketoconazole at 0.5 *μ*g·mL^−1^ concentration. Fluconazole sensitivity of 30.51% was observed at 32 *μ*g·mL^−1^ and itraconazole showed 7.55% inhibition at 16 *μ*g·mL^−1^. The MIC_50_ and MIC_90_ of fluconazole resistance strain were 34 and 47 *μ*g·mL^−1^, respectively, while itraconazole resistance strains showed MIC_50_ and MIC_90_ to be 36 and 49 *μ*g·mL^−1^, respectively.

**Figure 1:**
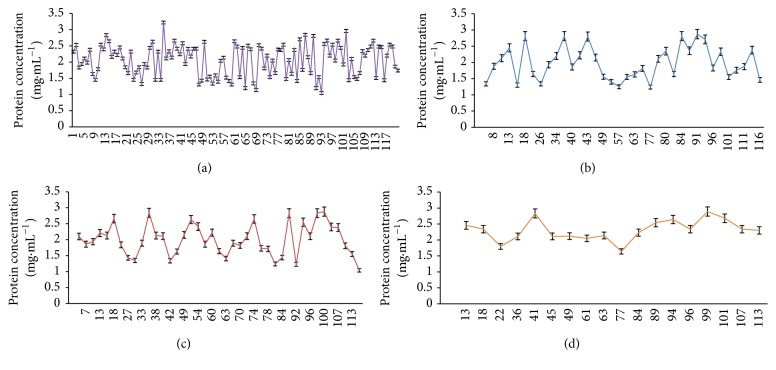
Protein concentration of drug resistant* C. albicans *isolated from high vaginal swab, catheter tip, and urine. Control (a), isolates showing itraconazole resistance (b), isolates showing fluconazole resistance (c), and isolates showing itraconazole and fluconazole resistance (d).

The total proteins were estimated using the method of Lowry et al. [12] which revealed the difference in the protein concentration (Figure 1) in a range from 1.05 to 3.22 mg·mL^−1^. When the organisms were growing in respective antifungal agent, there was decrease in the protein concentration in comparison to those isolates which were not subjected to any antifungal. It was also observed that all the strains which were having both fluconazole and itraconazole resistance were showing high protein concentration (>2 mg·mL^−1^) than the other resistant strains.

**Figure 2:**
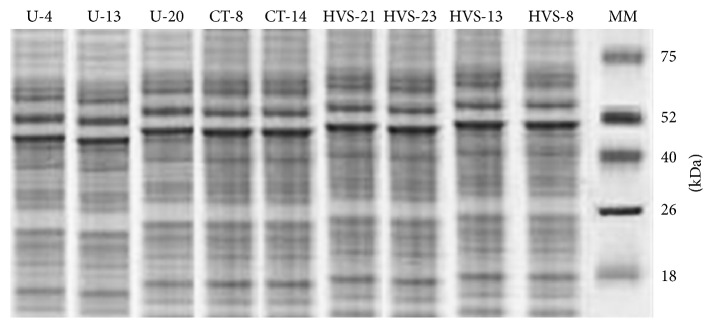
Protein profiling of fluconazole resistant* Candida albicans* isolated from high vaginal swab, catheter tip, and urine. The fluconazole resistant strains had a monomorphic thick band of ~52 kDa.

**Figure 3:**
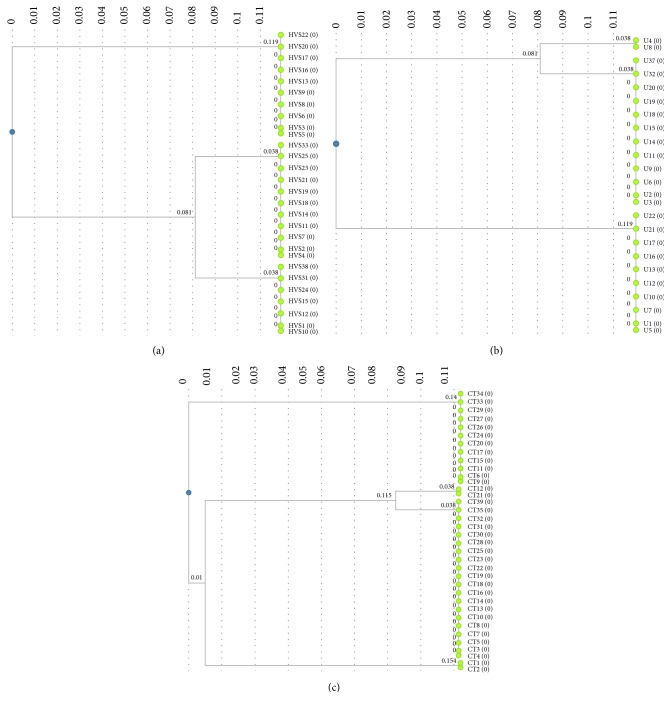
DendroUPGMA: relationship among fluconazole, itraconazole, and fluconazole and itraconazole resistant* Candida albicans *isolated from high vaginal swab (a), urine (b), and catheters tip (c) based on their protein profiles by SDS-PAGE.

When the strains were further analyzed for its difference in band pattern in SDS-PAGE, the fluconazole and itraconazole resistant strains were having approximately ~62.0 kDa and ~110 kDa similar molecular weight bands; moreover the fluconazole resistant strains were showing 86% polymorphic protein profiles apart from other strains. It was also observed that 72% of fluconazole resistant strains had a monomorphic thick band of ~52 kDa (Figure 2). Itraconazole resistant strains had lesser polymorphic similarity of 9.5% with lesser similarly of profile pattern. The polymorphic band pattern observed is summarized in Table 3. All the strains under study showed 22.73% of polymorphism. It was observed in all the three dendograms generated for the* Candida* isolated from high vaginal swab that urine and catheters tip had two clusters (Figure 3). Out of 28 antibiotic resistant high vaginal swab samples 10 strains with fluconazole resistance formed a separated cluster; moreover 7 strains with itraconazole and fluconazole resistance formed a separate cluster along with 11 strains having itraconazole resistance in cluster 2. The 24 antibiotic resistant urine* Candida* samples formed two clusters in which itraconazole and fluconazole resistance (2 strains) and itraconazole resistance (12 strains) were also clustered together. 36 antibiotic resistant isolates from catheters tip had also shown two clusters where 12 strains of itraconazole resistance formed a cluster and one itraconazole resistance strain showed similarity with the fluconazole resistance (23 strains). It was also observed that itraconazole and fluconazole resistance (2 strains) was clustered with fluconazole resistance strains in the case of catheter isolates. The present study showed similarity in protein profile of resistance strains indicating physiological variation in strains with respect to their antibiotic resistance pattern.

## 4. Discussion

Multidrug resistant candidiasis is an emerging thread, especially affecting women [13, 14]. It was observed that the catheterized patients were more susceptible to the* Candida* infections. There was considerable variation in drug resistance of* C. albicans *isolates from different sites. Decreased susceptibility to the azoles among clinical isolates of* C. albicans *has been earlier reported by [15]. Hadley et al. [16] and Hospenthal et al. [17] suggested that the drug susceptibility determination may contribute to better treatment of microbial infection. It was observed that the* C. albicans* susceptibility to antifungal agent is at increasing pattern [18].

The increase in the drug resistance is due to the widespread use of broad-spectrum antibiotics and steroids.* C. albicans *is considered as the most pathogenic member of the genus* Candida* which is the species most commonly isolated from clinical materials, although infections with other species of* Candida *have been reported [15, 19]. Mutations seen were associated with antifungal resistance in clinical isolates and these influenced morphogenesis acts as a key trait in the virulence.* C. albicans* diverse capacity to adapt to antifungal exposure [20] might be the reason for the increasing resistance that was observed in the study. Our study adds to the studies done on antifungal susceptibility in clinical strains of* C. albicans* which carries significance due to its increasing antifungal resistance and clinical importance as a threat of increased infection in immune compromised and hospital acquired infections. The MIC observations were comparable to the study of [21, 22] on the correlation of fluconazole MICs in* Candida *showed good 48 and 24 hr visual MICs in contrast to Espinel-Ingroff et al. [22] who evaluated 48 hr spectrophotometric and visual MICs of fluconazole, itraconazole, voriconazole, and posaconazole for Candida spp. This observation showed that spectrophotometric MICs are more objective than visual MICs. In a study similar to ours, Khosravi et al. [23] demonstrated that the use of SDS-PAGE showed a high degree of protein similarity among isolates of* C. albicans *with low and high virulence. Hence this evaluation can provide promising criteria for evolutionary studies in the increase of the serological changes in the drug resistance.

The SDS-PAGE pattern revealed several characteristic bands common to all isolates having similarity to the findings of Kobayashi and Suginaka [24] where the cell wall protein was used to distinguish serotypes in* C. albicans*. It was observed that the site of the sample had similarity in their total protein profile. The classification of the protein profile in the basis of virulence will help to evaluate the evolutionary difference in the antifungal resistance and constrain the resistance to antifungal in the fungi. The use of SDS-PAGE in epidemiological typing of nosocomial infection in neonates has been reported by Klebsiella spp. [6]. The efficacy of use of SDS-PAGE has also been described in aspergillosis in bronchogenic carcinoma [7]. Rodrigues et al. [8] reported the use of SDS-PAGE for typing of* C. albicans* isolates. They found this method to be important for characterizing the yeast for epidemiological and taxonomic purposes. Based on these findings, we evaluated the use of SDS-PAGE in* Candida albicans* isolates and performed analysis of the whole cell lysate to characterize the yeast. In the present study the fluconazole and itraconazole resistant strains showed 62 KDa and 110 KDa bands in SDS-PAGE so these strains were discussed. Jackson et al. [25] and Sagatova et al. [26] reported triazole drug resistant* C. albicans* has fungal lanosterol 14*α*-demethylase of 62 KDa. Song et al. [27] and Ying et al. [9] reported the ERG11 gene in fluconazole resistance of* C. albicans* and is an indicator for fluconazole resistance and has high content of ~50–65 kDa proteins of lanosterol 14 alpha-demethylase overexpression. The study clearly indicated that there is a difference in the total protein concentration with respective drug resistance, which can be used as a marker of the resistance development. The SDS-PAGE clearly indicated overexpression of the gene in the resistant strain. The protein isolated from the isolates cultivated with antifungal can be used to characterize and estimate the evolutionary scale in developing drug resistance.

## Figures and Tables

**Table 1:** Antifungal susceptibility patterns of clinical isolates.

Antifungal agent	Number of strains isolated from HVS, urine, and catheter tip	
	Sensitive	Resistant
Itraconazole	—	36 (35%)
Fluconazole	—	41 (50%)
Amphotericin B	120 (100%)	—
Ketoconazole	120 (100%)	—

**Table 2:** Minimum inhibitory concentration of clinical isolates.

Antifungal agent	Minimum inhibitory concentration (*µ*g·mL^−1^)							
	0.5	1	2	4	8	16	32	64
	Number of inhibited isolates							
Fluconazole	0	0	0	0	0	0	18	41
Itraconazole	0	0	0	0	0	4	13	36
Ketoconazole	13	120	—	—	—	—	—	—
Amphotericin B	22	120	—	—	—	—	—	—

**Table 3:** Banding pattern and molecular weight of clinical isolates.

Antifungal agent	Number of isolates	Polymorphic band (~kDa)	Number of bands
Fluconazole	41	22, 34, 75.5	23 (13.04%)
Itraconazole	36	13.5, 24, 105.5	18 (16.67%)
Fluconazole and itraconazole	11	12, 23, 54, 112	25 (16.00%)
Without antifungal agent	120	13, 16, 31, 54, 200	22 (22.73%)
